# High-risk human papillomavirus in non-melanoma skin lesions from renal allograft recipients and immunocompetent patients

**DOI:** 10.1038/bjc.2011.95

**Published:** 2011-03-22

**Authors:** M Reuschenbach, T Tran, F Faulstich, W Hartschuh, S Vinokurova, M Kloor, E Krautkrämer, M Zeier, M von Knebel Doeberitz, C Sommerer

**Affiliations:** 1 Department of Applied Tumor Biology, Institute of Pathology, University of Heidelberg, Im Neuenheimer Feld 220, Heidelberg 69120, Germany; 2 Clinical Cooperation Unit Applied Tumor Biology, German Cancer Research Center, Im Neuenheimer Feld 220, Heidelberg 69120, Germany; 3 Department of Nephrology, University of Heidelberg, Im Neuenheimer Feld 162, Heidelberg 69120, Germany; 4 Department of Dermatology, University of Heidelberg, Voßstraße 2, Heidelberg 69115, Germany

**Keywords:** human papillomavirus, non-melanoma skin cancer, renal transplantation, immunosuppression, p16^INK4a^

## Abstract

**Background:**

High-risk human papillomaviruses (HR-HPVs) can be detected in a proportion of non-melanoma skin cancers. Data on prevalence are inconclusive, but are essential to estimate the relevance of HR-HPV, particularly with regard to prophylactic HPV vaccines for skin cancer prevention.

**Methods:**

High-risk human papillomavirus DNA was investigated in 140 non-melanoma skin lesions from 54 immunocompetent patients and 33 immunosuppressed renal allograft recipients. Expression of p16^INK4a^, a marker for HR-HPV oncogene expression in the uterine cervix, and of p53 and pRB was evaluated immunohistochemically.

**Results:**

The highest prevalence of HR-HPV was found in squamous cell cancer (SCC) (46.2% (6 out of 13) in immunosuppressed and 23.5% (4 out of 17) in immunocompetent patients). High-risk human papillomavirus positivity was accompanied by diffuse p16^INK4a^ expression in most SCC (*P*<0.001) and basal cell cancers (*P*=0.02), while almost all SCC *in situ* were p16^INK4a^ positive irrespective of HR-HPV presence (*P*=0.66). Diffuse p16^INK4a^ expression was associated with lack of pRB expression (*P*=0.001). p53 was strongly expressed in 40.0% (56 out of 140) of the lesions irrespective of HR-HPV presence.

**Conclusion:**

High-risk human papillomavirus can be detected in lesions of keratinised squamous epithelia. The association of HR-HPV with diffuse p16^INK4a^ expression might indicate HR-HPV oncogene expression in a proportion of lesions. Overexpression of p53 suggests p53 pathway alterations in HR-HPV-positive and -negative lesions.

Non-melanoma skin cancer (NMSC) is the most frequent cancer type in fair-skinned populations and includes basal cell carcinoma (BCC) and squamous cell carcinoma (SCC) (Madan *et al*, 2010). The main risk factor is sun exposure, indicating that UV light-induced mutations have an important role in the carcinogenesis of NMSC (Ramos *et al*, 2004). Several other factors have been suggested to be involved in NMSC pathogenesis, in particular infectious agents (Nindl and Rosl, 2008). The increased incidence of NMSC in immunosuppressed compared with immunocompetent individuals (Grulich *et al*, 2007) suggests a central role of the immune system. In the general population, BCC accounts for approximately three fourths and SCC for one fourth of all NMSC cases. Compared with immunocompetent patients, the BCC/SCC ratio shifts towards SCC in renal transplant recipients, and the frequency of multiple, more aggressive lesions is high (Euvrard *et al*, 2003).

Basal cell carcinomas and SCCs of the skin are both keratinocyte-derived tumours which show marked differences in clinical and histopathological properties and most likely also in their aetiopathogenesis (Madan *et al*, 2010). Basal cell carcinomas are typically slow growing, locally invasive tumours that rarely metastasise (Rubin *et al*, 2005). By contrast, SCCs tend to be faster growing, locally invasive tumours with metastatic potential (Alam and Ratner, 2001). The molecular events taking place in BCC and SCC carcinogenesis are most likely heterogenic, and current data argue for a multi-step process frequently involving UV-induced *p53* mutations (Boukamp, 2005).

Human papillomavirus DNA of various genotypes has been detected in NMSC (de Villiers *et al*, 1997), but its role in NMSC carcinogenesis is unclear (Pfister, 2003; Nindl *et al*, 2007). In 30 to over 80% of NMSC and their precursor lesions, cutaneous and in particular epidermodysplasia verruciformis-associated HPV types have been detected (Forslund *et al*, 2003; Iftner *et al*, 2003; Nindl *et al*, 2004). Some studies report a higher HPV prevalence in lesions from immunosuppressed patients (Harwood *et al*, 2000; Stockfleth *et al*, 2004). Oncogenic high-risk HPV (HR-HPV) types, particularly HPV16, which are causally linked to the development of anogenital cancers and some head and neck cancers (zur Hausen, 2009), have been detected in 2–50% of NMSC and SCC *in situ* of the skin (Bowen's disease) (Harwood *et al*, 2000; Iftner *et al*, 2003; Nindl *et al*, 2004). Data on active viral oncogene expression and on differences in HR-HPV prevalence between immunosuppressed and immunocompetent patients are inconclusive.

Persistent HR-HPV infections can induce malignant transformation of mucosal squamous epithelial cells by inactivation of p53 and pRB once viral oncogenes *E6* and *E7* are expressed (Scheffner *et al*, 1990). High-risk human papillomavirus oncogene expression in cervical squamous epithelium is accompanied by strong nuclear and/or cytoplasmic diffuse overexpression of the cellular cyclin-dependent kinase inhibitor p16^INK4a^. In contrast, in transiently HR-HPV-infected cervical epithelia, in which HPV oncogene expression is low, no diffuse p16^INK4a^ expression is detectable (Sano *et al*, 1998; Klaes *et al*, 2001).

We here report evaluation of HR-HPV positivity in multiple invasive NMSC and non-invasive dysplastic epithelial skin lesions (SCC *in situ*, keratoacanthoma (KA)) from immunosuppressed renal allograft recipients and immunocompetent patients. High-risk human papillomavirus status was correlated with expression of p16^INK4a^, pRB, and p53. Gaining more insight into the expression status of cell cycle regulators in association with HR-HPV might help to estimate the relevance of HR-HPV in skin cancer tumour biology in general and particularly in transplant recipients with multiple non-melanoma skin lesions. This is of high relevance for estimating the benefit of prophylactic HPV vaccination for skin cancer prevention in the immunosuppressed. Besides viral infections, bacterial colonisation has attracted attention in cancer biology and might contribute to carcinogenesis particularly in immunocompromised conditions. Therefore, in addition to HR-HPV DNA prevalence, the presence of *Staphylococcus aureus* infection in the skin lesions was investigated. A significant association of *S. aureus* infection with SCC of the skin has been recently reported (Kullander *et al*, 2009). Although a causal role of *S. aureus* infection in development or progression of NMSC is not proven, the finding warrants further investigation, especially in multiple tumours from transplant recipients.

## Materials and methods

### Patients and tumour specimens

A consecutive collection of invasive NMSC and/or non-invasive non-melanoma epithelial skin lesions was obtained from renal allograft recipients treated in the Department of Nephrology at the University Hospital Heidelberg, Germany. Inclusion criteria were age >18 years, no history of autoimmune disease requiring additional immunosuppression, no history of other malignancies, at least one renal transplantation, regular follow-up visits at the renal outpatient clinic of the University Hospital Heidelberg, one or more invasive NMSC or non-invasive epithelial skin dysplasia located at non-anogenital sites diagnosed not earlier than 1 year after transplantation, and stable renal allograft function (estimated glomerular filtration rate (eGFR) >30 ml min^−1^). Invasive NMSC comprised SCC and BCC and non-invasive epithelial skin lesions comprised SCC *in situ* (also termed as Bowen's disease in the literature), KA, and actinic keratosis.

All renal allograft recipients had regular skin examination for malignoma and non-invasive epithelial skin lesions by a dermatologist at least once per year. Archival formalin-fixed, paraffin-embedded tissue of excised NMSC or non-invasive epithelial skin lesions was used in this study.

A cross-sectional cohort of patients with invasive NMSC or non-invasive epithelial skin lesions without known immunosuppression and with regular renal function (eGFR >80 ml min^−1^) served as controls. These patients were selected from the Department of Dermatology at the University Hospital Heidelberg based on the dermatopathology diagnosis to match the frequencies of different types of lesions with the immunosuppressed cohort.

The institutional Ethics Committee approved the protocol; informed consent was obtained from all enrolled patients.

### Microdissection and DNA extraction from tumour tissue

Formalin-fixed, paraffin-embedded tissue sections were stained with hematoxylin and eosin (H&E). Neoplastic tissue was morphologically identified under the light microscope and manually microdissected. DNA was extracted using the DNeasy Blood and Tissue kit (Qiagen, Hilden, Germany). The DNA was used for HPV testing and detection of *S. aureus*.

### Detection of HR-HPV DNA

High-risk human papillomavirus detection and genotyping were done using a multiplex test based on the Luminex technology (Multimetrix, Heidelberg, Germany) allowing for standardised detection and typing of the high-risk types HPV16, 18, 31, 33, 35, 39, 45, 51, 52, 56, 58, 59, 68, 73, and 82. PCR was performed using the supplied primers for amplification of HPV *L1* (Schmitt *et al*, 2006) and *β-globin* to ensure DNA integrity. From samples with negative *β-globin* amplification, DNA extraction was repeated from additional tumour sections to increase DNA yield. To monitor potential HR-HPV DNA contamination, formalin-fixed, paraffin-embedded tissues from colon adenomas were processed as negative controls throughout the complete procedure from tissue cutting, microdissection, DNA extraction, and genotyping.

### Detection of *S. aureus*

*Staphylococcus aureus* was detected by PCR amplification of the *S. aureus nuc* gene using previously published primers, forward 5′-GCGATTGATGGTGATACGGTT-3′ and reverse 5′-AGCCAAGCCTTGACGAACTAAAGC-3′, resulting in a 263-bp product (Brakstad *et al*, 1992; Kullander *et al*, 2009). PCR products were further analysed by DNA sequencing to confirm the *nuc* gene sequence.

### Immunohistochemistry for p16^INK4a^, pRB, and p53 expression

For immunohistochemical analyses, 2 *μ*m sections from formalin-fixed, paraffin-embedded tissues were used. p16^INK4a^ expression was detected using the CINtec Histology kit (mtm Laboratories, Heidelberg, Germany) according to the manufacturer's instructions. For p53 and pRB staining, antigen retrieval was performed in citrate buffer. Mouse monoclonal antibodies directed against p53 (clone DO-7, Dako, Glostrup, Denmark) and pRB (clone G3–245, BD Pharmingen, Franklin Lakes, NJ, USA) were used. Visualisation was done using the Vectastain Elite ABC kit (Vector Laboratories, Burlingame, CA, USA) and 3,3-diaminobenzidine (DAB) chromogen (Dako). The sections were counterstained with hematoxylin (Dako).

Lesions were categorised according to p16^INK4a^ expression as (a) positive for a diffuse pattern of nuclear and/or cytoplasmic p16^INK4a^ expression, beginning in the basal or parabasal layer and variably reaching intermediate and superficial cell layers, in analogy to squamous epithelial lesions at the uterine cervix (Klaes *et al*, 2001) and (b) negative for a diffuse p16^INK4a^ expression. Lesions classified as negative showed either a focal staining of single epithelial cells or were completely negative for p16^INK4a^ expression.

p53 and pRB expression of the lesions were categorised into three groups according to the proportion of tumour cells showing nuclear expression: (1) negative to very low (<10% of tumour cells), (2) moderate (10–50% of tumour cells), and (3) strong (>50% of tumour cells).

### Statistical data analysis

Results were calculated for all analysed skin lesions including all tumours from patients who had multiple tumours. Additionally, data from patients who had multiple skin lesions were examined separately. Parametric variables are given as mean±s.d., and non-parametric variables as median and range. Frequency distributions are provided for categorical variables. Fisher's exact test or Student's *t*-test was used to test the association between the parameters HPV status, p16^INK4a^/p53/pRB protein expression, immune status, age, gender, and sun exposure. Two-sided *P*-values <0.05 were considered as statistically significant. Associations were tested for separate strata by Breslow–Day test for homogeneity of odds ratios and Chochran–Mantel–Haenszel test for conditional independence. An association was considered independent from the applied strata when Breslow–Day test and Chochran–Mantel–Haenszel test retrieved *P*-values >0.05. Data analyses were carried out using SPSS (v16.0) software (SPSS, Munich, Germany).

## Results

### Patient and skin lesion characteristics

**Figure 1:**
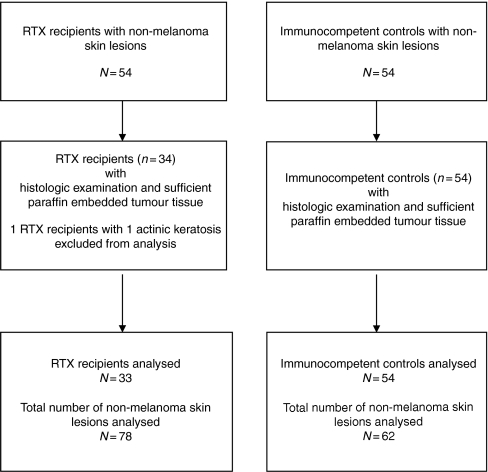
Study flow chart. RTX=renal transplantation.

Fifty-four renal allograft recipients with non-melanoma skin lesions recorded in the clinical database fulfilled the inclusion criteria. From 34 patients (25 male, 9 female; median age of 67 (44–75) years) with a total number of 79 non-melanoma skin lesions, tumour tissue was available. In addition, 62 non-melanoma skin lesions from 54 immunocompetent controls (36 male, 18 female; median age 71 (43–87) years) were included in the present study (Table 1; Figure 1). All immunosuppressed and immunocompetent patients were Caucasian and had skin type Fitzpatrick II or III.

One patient with actinic keratosis was excluded from further analyses. Histology evaluation of the 78 skin biopsies of the remaining 33 immunosuppressed patients revealed 13 invasive SCC, 35 BCC, 24 SCC *in situ*, and 6 KA. From 54 immunocompetent controls, 17 invasive SCC, 18 BCC, 17 SCC *in situ*, and 10 KA were analysed. Lesions diagnosed as KA were non-invasive dysplastic squamous cell alterations of the skin. The proportion of lesions affecting sun-exposed skin areas (head and hands) was 78.8% in the immunocompetent compared with 59.5% in the immunosuppressed patients.

**Figure 2:**
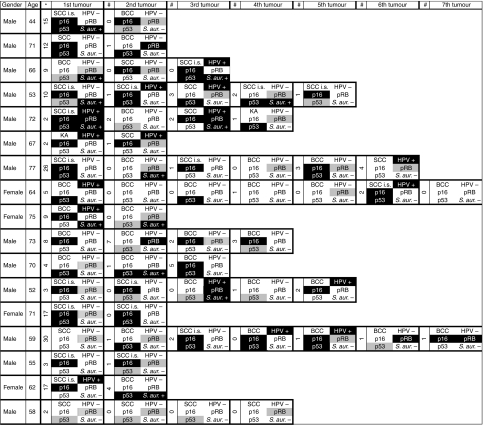
Results of non-melanoma skin lesions from immunosuppressed patients with multiple tumours. Age is given at occurrence of first skin lesion. ^*^Time (years) between first transplantation and first skin lesion. #Time (years) between occurrence of next skin lesion. Black background indicates positive results for HPV/*S. aureus* or diffuse/strong expression of p16^INK4a^ (diffuse)/pRB (strong)/p53 (strong). Grey background indicates moderate expression of pRB/p53. White background indicates negativity. SCC=squamous cell cancer; BCC=basal cell cancer; KA=keratoacanthoma; SCC i.s.=SCC *in situ*; p16=p16^INK4a^; *S. aur.*=*Staphylococcus aureus*.

Median time of non-melanoma skin lesion occurrence in renal transplant recipients was 10.5 years (1–38) after transplantation (SCC 6 years (1–34), BCC 9 years (2–36), SCC *in situ* 13 years (2–33), and KA 15 years (1–30)). Primary immunosuppression and immunosuppressive therapy at the time of the first non-melanoma skin lesion is included in Table 1. No association between type of non-melanoma skin lesion and immunosuppressive treatment could be detected in the present patient cohort. In all, 51.5% (17 out of 33) immunosuppressed patients had multiple skin lesions (9 with 2 or 3 lesions, 6 with 4–6 lesions, and 2 with 7 lesions) which occurred either synchronously (26 out of 62 lesions) or metachronously (36 out of 62 lesions, median time between occurrence of lesions 1.5 years (1–7), included in Figure 2). Most of the patients (11 out of 17, 64.7%) with multiple skin lesions had a history of azathioprine therapy. In the cohort of immunocompetent patients, eight patients had two synchronous lesions.

### Frequency of HR-HPV

Among all 140 analysed skin lesions including lesions from patients with multiple tumours, mucosal HR-HPV DNA was detected predominantly in SCC *in situ* and SCC with 39.0% (16 out of 41) of SCC *in situ* and 33.3% (10 out of 30) of SCC. In all, 20.5% (9 out of 44) of BCC and 6.3% (1 out of 16) of KA tested HR-HPV positive.

Human papillomavirus DNA was detected in 29.5% (23 out of 78) of skin lesions from immunosuppressed and in 20.9% (13 out of 62) lesions from immunocompetent individuals (Fisher's exact *P*=0.171). Stratification by the dermatopathology diagnosis indicated a comparable HR-HPV prevalence between immunosuppressed and immunocompetent patients in SCC *in situ* and BCC, but a slightly higher prevalence of HR-HPV in SCC from the immunosuppressed patients (46.2% (6 out of 13) *vs* 23.5% (4 out of 17)) (Table 2). High-risk human papillomavirus status in association with immune status was independent from the strata age, gender, and sun exposure. All HR-HPV-positive lesions were genotyped as positive for HPV16. Multiple infections with HR-HPV types 18, 39, 45, and/or 56 together with HPV16 were detected in seven lesions from immunosuppressed patients (three SCC *in situ*, two SCC, and two BCC) and in one lesion from the immunocompetent group (one SCC) (Table 2).

Most (52.9%, 9 out of 17) immunosuppressed patients with multiple skin lesions had HPV-negative and HPV-positive tumours. In all, 35.3% (6 out of 17) had only HPV-negative lesions (Figure 2). One immunocompetent patient with two lesions had one HPV-negative and one HPV-positive lesion. The remaining immunocompetent patients with two lesions (87.5%, 7 out of 8) had HPV-negative lesions only.

### p16^INK4a^ expression and correlation with HR-HPV

**Figure 3:**
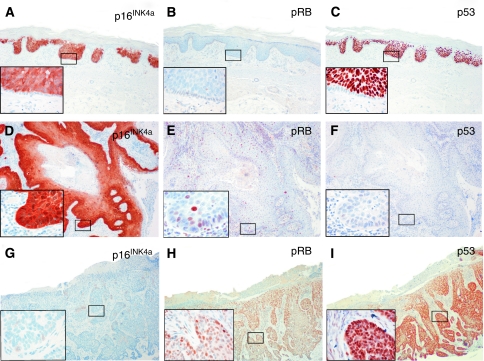
Immunohistochemical staining of serial tissue sections from non-melanoma skin lesions for p16^INK4a^, pRB, and p53. (**A**–**C**) HR-HPV-positive SCC *in situ* with diffuse p16^INK4a^ expression pattern (**A**), absence of pRB expression (**B**), and strong p53 expression (**C**). Note the absence of p16^INK4a^, pRB, and p53 expression in the basal cell layer of this SCC *in situ*. (**D**–**F**) HR-HPV-positive invasive SCC with diffuse p16^INK4a^ expression pattern (**D**), weak pRB expression (**E**), and absence of p53 expression (**F**). (**G**–**I**) HR-HPV-negative BCC with absence of diffuse p16^INK4a^ expression, moderate pRB expression, and strong p53 expression.

A diffuse, continuous pattern of cytoplasmic and/or nuclear p16^INK4a^ expression arising from basal and parabasal epithelial cells as it is characteristic for HR-HPV-transformed mucosal squamous epithelia was observed in 47.1% (66 out of 140) of skin lesions, with the highest frequency in SCC *in situ* (92.7%, 38 out of 41). Most SCC *in situ* showed diffuse p16^INK4a^ expression beginning in the parabasal cell layer, but no p16^INK4a^ expression in the basal cell layer (Figure 3); 36.7% (11 out of 30) of the invasive SCC, 30.2% (16 out of 53) of the BCC, and 6.7% (1 out of 15) KA showed a diffuse p16^INK4a^ expression pattern (Table 2; Figure 3). Altogether, 86.1% (31 out of 36) of HR-HPV-positive lesions demonstrated a diffuse p16^INK4a^ expression pattern compared with 33.7% (35 out of 104) of HR-HPV-negative lesions (*P*<0.001). In almost all SCC, BCC, and KA lesions with a diffuse p16^INK4a^ expression pattern, HPV DNA was detected, whereas nearly all SCC *in situ* were p16^INK4a^ positive, irrespective of HR-HPV status (see Table 3 for detailed results).

### pRB and p53 expression and relation to HR-HPV and p16^INK4a^

Frequency of pRB expression is provided in Table 2. In most of the non-melanoma skin lesions positive for a diffuse p16^INK4a^ expression pattern (*n*=66), no pRB expression was detectable (50 negative, 13 moderate, and 3 strong; *P*=0.001).

Strong p53 expression was detected in 40.0% (56 out of 140) of the analysed skin lesions and particularly frequent in SCC *in situ* (65.9%, 27 out of 41). The frequency of strong p53 expression was comparable between immunosuppressed and immunocompetent patients in SCC *in situ* and BCC, while in invasive SCC the frequency of a strong p53 expression was lower in immunosuppressed than in immunocompetent patients (7.7% (1 out of 13) *vs* 41.2% (7 out of 17), *P*=0.04; Table 2). No association between p53 and p16^INK4a^ expression was observed. In all, 25.7% of all lesions showed both, diffuse p16^INK4a^, and strong p53 expression; 21.4% showed a diffuse p16^INK4a^ expression pattern while lacking strong p53 expression; 14.3% showed strong p53 expression, but no diffuse p16^INK4a^ expression pattern.

Tumours from patients with multiple lesions showed heterogeneity for HR-HPV status, p16^INK4a^, pRB, and p53 expression (Figure 2).

### *Staphylococcus aureus* infection

*Staphylococcus aureus* was detected in 30.6% (19 out of 62) non-melanoma skin lesions from immunocompetent and in 26.9% (21 out of 78) from immunosuppressed patients (*P*=0.38). Altogether, 30.0% (9 out of 30) SCC, 18.9% BCC (10 out of 53), 30% SCC *in situ* (16 out of 41), and 45.5% (5 out of 11) KA were positive for *S. aureus* (Table 2).

## Discussion

Human papillomavirus DNA, including HR-HPV types known to cause anogenital and a proportion of head and neck cancers, has been repeatedly detected in NMSC (de Villiers *et al*, 1997) and non-invasive epithelial skin lesions. From studies in anogenital and head and neck cancers it is well known that HR-HPV oncogenes, once they are expressed, interact with cellular cell cycle regulators and contribute to uncontrolled cell proliferation, most importantly by degradation of pRB and p53 (Scheffner *et al*, 1990). p16^INK4a^ is expressed strongly and continuously in cells undergoing HR-HPV-mediated transformation in epithelia of the uterine cervix. Though the mechanism is not fully clear, there is evidence that a negative feedback mechanism controlling p16^INK4a^ levels in normal cells is disrupted by a reduction of pRB activity in cells expressing HR-HPV oncogene *E7* (Khleif *et al*, 1996). Also some other cancer types, irrespective of the HPV status, show an inverse correlation between pRB and p16^INK4a^ expression levels (Okamoto *et al*, 1994; Geradts *et al*, 1995).

In the present systematic explorative study, we demonstrated that mucosal HR-HPV DNA (HPV16) can be detected in keratinised squamous epithelium, at a frequency of 23–46% in analysed SCC *in situ* and invasive SCC and 11–20% in BCC. This finding is in line with previous reports, although frequencies varied between 2% and 50%, which is most likely due to different HPV detection methods (Harwood *et al*, 2000; Iftner *et al*, 2003; Nindl *et al*, 2004).

We observed a strong correlation between HR-HPV positivity and diffuse p16^INK4a^ expression in invasive SCC and BCC. Most p16^INK4a^-positive lesions lacked pRB expression. Although this observational study cannot formally prove a transforming role of HR-HPV, the finding of frequent pRB absence and p16^INK4a^ overexpression in HR-HPV-positive SCC and BCC might suggest a potential relevance of HR-HPV in NMSC. Basal epithelial cells in SCC *in situ* often lacked p16^INK4a^ and p53 expression. This finding might be compatible with the assumption that the basal cells are most likely not involved in SCC *in situ* carcinogenesis (Ishida *et al*, 2001). Although HR-HPV could be detected in a proportion of SCC *in situ*, mechanisms other than HR-HPV might cause a non-functional pRB pathway and thus be responsible for p16^INK4a^ overexpression and absence of pRB expression.

Strong p53 expression was observed in 40% of the analysed tumours irrespective of HPV status. This observation might suggest presence of mutations in the *p53* tumour suppressor gene in these tumours, as p53 overexpression has been linked to certain *p53* mutations (Benjamin *et al*, 2008; Kusters-Vandevelde *et al*, 2010). However, a high level of detectable p53 protein has also been observed without detectable p53 mutations (Stark *et al*, 1994).

There is evidence from the literature that p53 overexpression (and *p53* mutations) and HR-HPV positivity are inversely correlated in oropharyngeal and vulvar cancer, which led to the classification of molecularly distinct tumour entities (HR-HPV positive/p53 expression negative and HR-HPV negative/p53 expression positive) that also show marked differences in their clinical behaviour (Kruse *et al*, 2008; Smith *et al*, 2008), though p53 has also been found strongly expressed in some HR-HPV transformed cancer cells (Cheah and Looi, 2002). Based on our data, there is no correlation between p53 expression and HR-HPV status in epithelial skin lesions. Thus, it may well be that in skin carcinogenesis, the HR-HPV oncoprotein-mediated degradation of p53 is not as important as in the pathogenesis of known HR-HPV-associated cancers and *p53* mutations or other genetic/epigenetic alterations have a primary role in skin carcinogenesis. HPV might then be an additional factor contributing to malignant transformation of the skin. Interestingly, we and others (Blokx *et al*, 2003; Kusters-Vandevelde *et al*, 2009) found a lower frequency of p53 overexpression in invasive SSC from immunosuppressed compared with immunocompetent patients. The biological and clinical relevance of this finding needs to be addressed on larger patient cohorts.

The status of p16^INK4a^, pRB, and p53 expression was discordant among multiple tumours from the same patient, suggesting varying carcinogenic modes or stages. Inter-tumoural diversity was also found regarding HR-HPV status – most patients with multiple tumours had both, HR-HPV-positive and -negative tumours. Nevertheless, clonality of HR-HPV-positive tumours should be assessed in future studies.

Besides viral infection, bacterial colonisation of the skin might contribute to a higher incidence of skin lesions in the immunosuppressed. The prevalence of *S. aureus* has been reported to be higher in skin SCC than in BCC or normal skin (Kullander *et al*, 2009). In our cohort, *S. aureus* DNA was detected in over 30% of invasive SCC and SCC *in situ* and, less frequently, in BCC. This also applied to the group of immunosuppressed patients. Presence of *S. aureus* in NMSC might simply indicate an increased susceptibility for bacterial colonisation of neoplastically altered skin. On the other hand, a pathogenetic role of *S. aureus* cannot be excluded, as bacteria might contribute to carcinogenesis by inducing chronic inflammation.

In contrast to the higher prevalence of SCC than BCC reported for transplant recipients (Euvrard *et al*, 2003), the prevalence of BCC was higher than SCC in our cohort. Also, patients who were excluded from the study were more likely to have BCC than SCC (data not shown). Regular systematic dermatology evaluations and non-surgical therapy for keratotic skin lesions might explain the relatively low prevalence of invasive SCC in our cohort. Moreover, in our hospital an early low-dose calcineurin inhibitor regimen has been established which might affect tumour prevalence in so far as high-dose calcineurin inhibitor regimens are supposed to increase the risk of especially SCC (Wu *et al*, 2010).

In summary, we conclude that HR-HPV DNA is detectable in keratinised skin and might contribute to skin cancer pathogenesis in a proportion of lesions. High-risk human papillomavirus prevalence in invasive skin SCC tended to be slightly higher in immunosuppressed than in immunocompetent patients. Further studies are warranted on the relevance of HPV in NMSC carcinogenesis, especially with regard to a potential beneficial effect of prophylactic HPV vaccines in skin cancer prevention, which would be particularly important in organ transplant recipients.

## Figures and Tables

**Table 1:** Demographics of renal allograft recipients (immunosuppressed) and immunocompetent patients (median, minimum, and maximum; number and percentage)

	**Renal transplant recipients (*N*=33)**	**Immunocompetent controls (*N*=54)**
Age (years)	67 (44–78)	71 (43–87)
Gender (male, %)	24 (72.7)	37 (68.5)
Time since transplantation (years)	10.5 (1–38)	—
Duration of haemodialysis (years)	3 (0–15)	—
		
*Renal disease (number,* %)		—
Diabetes	3 (9.1)	
Hypertension	3 (9.1)	
Glomerulonephritis	16 (48.5)	
ADPKD	4 (12.1)	
Others	7 (21.2)	
		
*Transplantation*		—
First (number, %)	26 (78.8)	
Second (number, %)	6 (18.2)	
Third (number, %)	1 (3.0)	
		
*Renal allograft function*		
Creatinine (mg dl^−1^)	1.18 (0.63–2.29)	
		
*Immunosuppression at time of transplantation/ at time of first non-melanoma skin lesion*		
Ciclosporin A+Azathioprin+Steroids	12/4	
Azathioprin+Steroids	5/1	
Ciclosporin A+Steroids	5/16	
Ciclosporin A+MMF+Steroids	9/9	
Tacrolimus+MMF+Steroids	2/3	

Abbreviations: ADPKD=autosomal dominant polycystic kidney disease; MMF=mycophenolate mofetil.

**Table 2:** Results of HR-HPV detection, p16^INK4a^, pRB, p53 expression analysis, and *Staphylococcus aureus* detection in all 140 analysed skin lesions from renal transplant recipients and immunocompetent patients

	**Lesions from renal transplant recipients**								**Lesions from immunocompetent patients**							
	**SCC *in situ***		**SCC**		**BCC**		**KA**		**SCC *in situ***		**SCC**		**BCC**		**KA**	
	***n***	**%**	***n***	**%**	***n***	**%**	***n***	**%**	***n***	**%**	***n***	**%**	***n***	**%**	***n***	**%**
**Total *n***	**24**		**13**		**35**		**6**		**17**		**17**		**18**		**10**	
*HR-HPV*																
Negative	15	62.5	7	53.8	28	80.0	5	83.3	10	58.8	13	76.5	16	88.9	10	100.0
Positive	9	37.5	6	46.2	7	20.0	1	16.7	7	41.2	4	23.5	2	11.1	0	0.0
1 HPV type	6	25.0	4	30.8	5	14.3	1	16.7	7	41.2	3	17.6	2	11.1	0	0.0
2–3 HPV types	1	4.2	2	15.4	2	5.7	0	0.0	0	0.0	0	0.0	0	0.0	0	0.0
>3 HPV types	2	8.3	0	0.0	0	0.0	0	0.0	0	0.0	1	5.9	0	0.0	0	0.0
																
*p16* ^*INK4a*^																
No expression	1	4.2	0	0.0	3	8.6	1	16.7	2	11.8	1	5.9	0	0.0	0	0.0
Focal	0	0.0	7	53.8	22	62.9	4	66.7	0	0.0	11	64.7	12	66.7	10	100.0
Diffuse	23	95.8	6	46.2	10	28.6	1	16.7	15	88.2	5	29.4	6	33.3	0	0.0
																
*pRB*																
No expression	18	75.0	6	46.2	15	42.9	1	16.7	14	82.4	8	47.1	13	72.2	7	70.0
Moderate	5	20.8	7	53.8	12	34.3	4	66.7	2	11.8	9	52.9	5	27.8	3	30.0
Strong	1	4.2	0	0.0	8	22.9	1	16.7	1	5.9	0	0.0	0	0.0	0	0.0
																
*p53*																
No expression	5	20.8	6	46.2	12	34.3	2	33.3	3	17.6	4	23.5	9	50.0	8	80.0
Moderate	3	12.5	6	46.2	10	28.6	3	50.0	3	17.6	6	35.3	2	11.1	2	20.0
Strong	16	66.7	1	7.7	13	37.1	1	16.7	11	64.7	7	41.2	7	38.9	0	0.0
																
*Staphylococcus aureus*																
Negative	16	16.7	9	69.2	27	77.1	5	83.3	9	52.9	12	70.6	16	88.9	6	60.0
Positive	8	33.3	4	30.8	8	22.9	1	16.7	8	47.1	5	29.4	2	11.1	4	40.0

Abbreviations: SCC=squamous cell cancer; BCC=basal cell cancer; KA=keratoacanthoma; HR-HPV=high-risk human papillomavirus.

**Table 3:** HR-HPV infection and diffuse p16^INK4a^ expression pattern in non-melanoma skin lesions from renal transplant recipients and immunocompetent controls

	**Renal transplant recipients NMSC lesions (*N*=78)**					**Immunocompetent controls NMSC lesions (N=62)**				
	**HR-HPV+**		**HR-HPV−**			**HR-HPV+**		**HR-HPV−**		
	**p16^INK4a^+**	**p16^INK4a^−**	**p16^INK4a^ +**	**p16^INK4a^−**	***P***	**p16^INK4a^+**	**p16^INK4a^−**	**p16^INK4a^+**	**p16^INK4a^−**	***P***
SCC	5	1	1	6	<0.05	4	0	1	12	<0.001
BCC	4	3	6	22	0.06	2	0	4	12	<0.05
SCC *in situ*	9	0	14	1	NS	6	1	9	1	NS
KA	1	0	0	5	<0.05	0	0	0	10	—
All lesions	19	4	21	34	<0.001	12	1	14	35	<0.001

Abbreviations: SCC=squamous cell cancer; BCC=basal cell cancer; KA=keratoacanthoma; HR-HPV=high-risk human papillomavirus; NMSC=non-melanoma skin cancer.
